# Deep learning in multi-omics integration for gastrointestinal cancer biomarker discovery

**DOI:** 10.3389/fonc.2026.1856641

**Published:** 2026-07-01

**Authors:** Jing Lv, Deyin Liu, Lei Guo, Yangyang Wang, Jihan Wang, Jing Huang

**Affiliations:** 1 Department of Clinical Laboratory, Honghui Hospital, Xi’an Jiaotong University, Xi’an, Shaanxi, China; 2 Department of Trauma Orthopaedics, Honghui Hospital, Xi’an Jiaotong University, Xi’an, Shaanxi, China; 3 Department of Spine Surgery, Honghui Hospital, Xi’an Jiaotong University, Xi’an, Shaanxi, China; 4 School of Physics and Electronic Information, Yan’an University, Yan’an, Shaanxi, China; 5 Yan’an Medical College of Yan’an University, Yan’an, Shaanxi, China; 6 Department of Clinical Pharmacy, Honghui Hospital, Xi’an Jiaotong University, Xi’an, Shaanxi, China

**Keywords:** artificial intelligence, biomarker discovery, deep learning, gastrointestinal cancers, multi-omics

## Abstract

Gastrointestinal (GI) cancers remain leading causes of cancer-related morbidity and mortality worldwide, driven by complex molecular mechanisms involving genomic, transcriptomic, proteomic, metabolomic, and radiomic alterations. The discovery of reliable biomarkers is essential for improving early detection, risk stratification, prognosis prediction, and personalized treatment strategies. Recent advances in deep learning (DL) have enabled the integration of multi-omics and imaging data, offering unprecedented opportunities to identify clinically relevant biomarkers in these malignancies. This review synthesizes representative DL architectures specifically optimized for GI cancer biomarker research, highlighting methodological advances in feature extraction, multi-modal data fusion, and predictive modeling. Beyond detailing technical architectures, we critically examine current challenges, including the reliance on public datasets, the impact of intratumoral heterogeneity, and the need for rigorous external validation. We further discuss emerging strategies such as biologically-informed deep learning and multimodal foundation models for enhancing model interpretability, addressing data heterogeneity, and supporting translational adoption within regulatory frameworks (e.g., FDA/NMPA). By bridging computational innovations with clinical needs, DL-enabled multi-omics integration holds promise for advancing predictive, preventive, and personalized medicine in gastrointestinal oncology.

## Introduction

Gastrointestinal (GI) cancers, including esophageal, gastric colorectal, hepatocellular, and pancreatic carcinomas, represent a major global health burden, accounting for a substantial proportion of cancer-related morbidity and mortality worldwide (1, 2). Despite clinical advancements, the early detection and accurate prognosis of these malignancies remain challenging due to the limitations of conventional diagnostic methods in terms of sensitivity and specificity (3, 4). Biomarkers have emerged as pivotal tools for improving early diagnosis, prognostic stratification, and personalized treatment strategies (5, 6). However, the biological complexity and intertumoral heterogeneity of GI cancers pose significant obstacles to reliable biomarker discovery (7, 8).

Currently, a significant gap exists between the molecular understanding of GI cancers and clinical practice. For instance, while microsatellite instability (MSI) is a well-established biomarker in colorectal cancer (CRC), valid predictive biomarkers for chemotherapy response in gastric and pancreatic cancers remain scarce. Traditional single-omics approaches often fail to capture the systemic dysregulation characterizing these tumors.

The advent of multi-omics technologies has revolutionized our understanding of GI cancer pathogenesis. By integrating genomics, transcriptomics, proteomics, metabolomics, and radiomics, researchers can now obtain a multidimensional perspective of tumor biology (9, 10). Genomic and transcriptomic methods provide insight into genetic alterations and dysregulated gene expression (11), whereas proteomics and metabolomics methods reveal functional perturbations in protein networks and metabolic pathways (12, 13). Multimodal imaging analysis, encompassing macro-scale radiomics from medical scans and micro-scale pathomics from histopathological slides, enables non-invasive and high-throughput characterization of tumor heterogeneity (14, 15). The convergence of these omics layers offers unprecedented opportunities for biomarker identification and precision oncology (16, 17).

However, multi-omics integration presents formidable challenges, including high dimensionality, data heterogeneity, and complex cross-omics interactions. Traditional analytical methods often fail to capture these complex relationships (18). Deep learning (DL), a transformative branch of artificial intelligence (AI), offers potential in handling large-scale, high-dimensional biomedical data (19). Through deep neural networks (DNNs), DL models are capable of representing latent biological patterns, identifying nonlinear feature correlations, and increasing predictive accuracy in biomarker research (20). Architectures such as convolutional neural networks (CNNs) (21, 22), recurrent neural networks (RNNs), graph neural networks (GNNs) (23), autoencoders, and transformer models have demonstrated exceptional potential in multi-omics analysis (24–26).

While several recent reviews have discussed AI in oncology in general terms, there is a lack of synthesis specifically focused on the unique multi-modal challenges of GI cancers—such as the integration of endoscopic/radiological imaging with molecular profiles. Unlike previous works that function as general DL tutorials, this review specifically critically evaluates DL architectures through the lens of GI cancer applications. We highlight how specific architectures address the high dimensionality of omics data and the spatial heterogeneity of GI tumors, while rigorously addressing the pitfalls of current datasets, such as reliance on The Cancer Genome Atlas (TCGA), intratumoral heterogeneity, and the translational gap between in silico models and clinical decision-support systems. By synthesizing current advancements and outlining future directions, this review underscores the paradigm-shifting potential of DL techniques in advancing multi-omics-driven precision oncology for GI cancers.

## Literature search methodology

To ensure a comprehensive and rigorous review, we conducted a systematic literature search using the PubMed, Web of Science, and Scopus databases. The search covered the period from 2017 to 2025 to capture the most recent advancements in deep learning. Keywords included combinations of (“Deep Learning” OR “Artificial Intelligence”) AND (“Multi-omics” OR “Genomics” OR “Transcriptomics” OR “Epigenomics” OR “Proteomics” OR “Metabolomics” OR “Radiomics”) AND (“Gastrointestinal Cancer” OR “Colorectal Cancer” OR “Gastric Cancer” OR “Hepatocellular Carcinoma/Liver Cancer” OR “Pan-cancer”). Studies were included if they (1): applied DL techniques; and (2) focused specifically on GI malignancies or involved pan-cancer analyses with distinct findings relevant to GI cancers.

## Deep learning techniques for multi-omics data integration

The integration of multi-omics data offers unprecedented opportunities to elucidate the complex molecular landscape of GI cancers. However, the inherent challenges of data heterogeneity, high dimensionality, and complex biological interactions necessitate advanced computational approaches (21, 27, 28). DL techniques have emerged as a transformative solution because of their exceptional capacity for automated feature extraction and pattern recognition in complex datasets (29–31). This section systematically reviews fundamental DL architectures and their innovative applications in multi-omics and radiomics integration for GI cancer biomarker discovery (12).

### Overview of deep learning architectures

**Figure 1:**
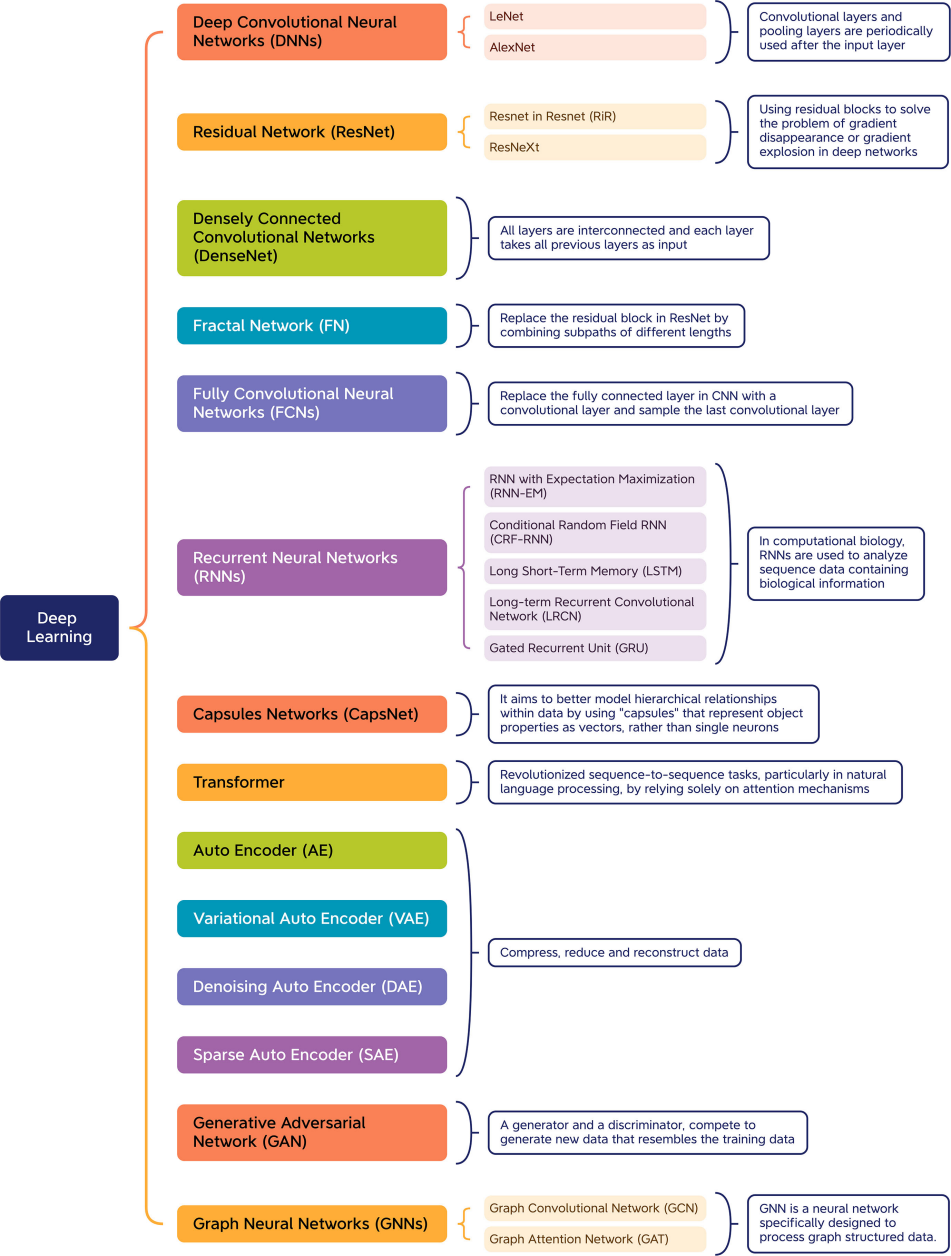
Schematic representation of deep learning architectures for multi-omics data analysis. Convolutional networks (CNNs, ResNet, DenseNet, FCNs): Primarily employed for extracting spatial features from radiological images (radiomics) and histopathological slides (pathomics). Recurrent networks (RNNs, LSTM, GRU): utilized for modeling sequential data, such as genomic sequences, longitudinal clinical records, or time-series metabolomic profiles. Transformers: Leverage attention mechanisms to capture global dependencies across heterogeneous data modalities, increasingly replacing RNNs for analyzing complex molecular sequences. Autoencoders (AE, VAE, DAE, SAE): Serve as unsupervised engines for dimensionality reduction and noise filtering in sparse high-throughput omics data (e.g., scRNA-seq). Graph neural networks (GNNs): Designed to model non-Euclidean data structures, such as protein–protein interaction networks and metabolic pathways, identifying key regulatory hubs in GI tumorigenesis.

To address the unique characteristics of multi-omics data, various DL architectures have been adapted to capture distinct biological relationships (**Figure 1**). CNNs, originally capable of spatial pattern recognition, are extensively used to extract high-level features from macro-scale radiomics data (e.g., CT/MRI) and micro-scale pathomics data (e.g., whole-slide images), as well as identify regulatory motifs in genomics (11, 14, 21). For longitudinal or sequential data, (RNNs and long short-term memory (LSTM) networks excel at modeling temporal dependencies (20, 32), whereas Transformer models utilize self-attention mechanisms to capture global dependencies across large datasets more efficiently than sequential models (15, 33). To manage high dimensionality, Autoencoders (AEs) and variational Autoencoders (VAEs) offer unsupervised frameworks for dimensionality reduction and noise filtering (13, 34). Furthermore, GNNs are uniquely powerful for encoding relational dependencies within complex biological networks, such as protein–protein interactions and metabolic pathways (16). The selection between these architectures often hinges on the data’s structural properties: while GNNs are optimized for capturing the explicit topological constraints of biological networks, Transformers excel at identifying latent, long-range correlations within high-dimensional sequences. Collectively, these architectures provide a robust toolkit for deciphering the multi-layered complexity of GI cancers.

### Methods for multi-omics data integration in deep learning

**Figure 2:**
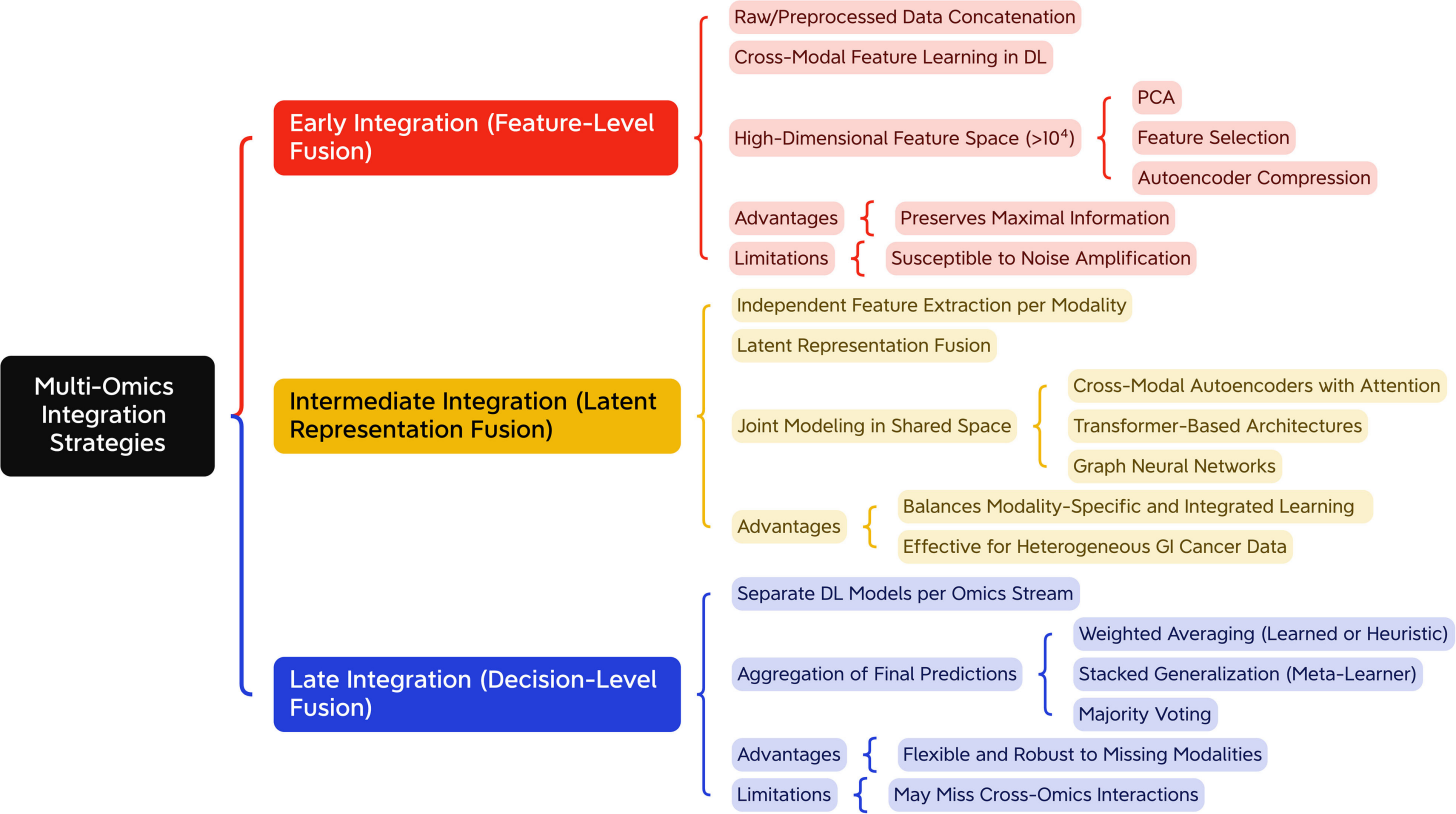
Schematic overview of deep learning-based multi-omics data integration strategies.

DL approaches for multi-omics integration can be systematically classified into three principal strategies based on their fusion stage: early (feature-level), intermediate (latent representation), and late (decision-level) integration. Each paradigm offers distinct advantages and computational considerations for biomarker discovery in GI cancers. **Figure 2** shows a schematic overview of the multi-omics data integration methodologies employed in DL applications.

Early integration (feature-level fusion): Early integration approaches combine raw or preprocessed multi-omics data through direct concatenation prior to model input, enabling DL architectures to learn cross-modal feature interactions from initial processing stages. While this approach preserves maximal information content, the resulting high-dimensional feature space (often exceeding tens of thousands of dimensions) presents significant computational challenges. Common solutions include dimensionality reduction techniques such as principal component analysis (PCA), feature selection algorithms, and autoencoder-based compression (5). This strategy is particularly effective when strong inter-omics correlations exist, although it may be susceptible to noise amplification from uncurated feature spaces.

Intermediate integration (latent representation fusion): Intermediate integration approaches employ a more advanced hierarchical approach where (1) each omics modality undergoes independent feature extraction (2), learned latent representations are fused in a shared embedding space, and (3) joint modeling occurs through downstream neural network layers. State-of-the-art implementations utilize cross-modal autoencoders with attention gates, transformer-based architectures for context-aware fusion, and GNNs for biological network integration (23). This balanced approach maintains modality-specific feature learning while enabling biologically meaningful cross-omics interactions, making it particularly valuable for heterogeneous GI cancer datasets.

Late integration (decision-level fusion): Late integration approaches adopt a modular framework where (1) separate DL models process each omics stream independently, and (2) final predictions are aggregated through ensemble techniques. Common fusion methods include weighted averaging (learned or heuristic), stacked generalization (meta-learner), and majority voting schemes (20). This strategy offers computational flexibility in incorporating heterogeneous data sources and robustness to missing modalities, although it may overlook important cross-omics interactions present in earlier fusion approaches.

The optimal integration strategy depends on multiple factors, including data characterization (dimensionality, noise level, and missingness), computational resources, biological hypotheses (focused vs. exploratory), and clinical application requirements. Emerging hybrid approaches that combine elements from multiple integration levels show particular promise for GI cancer applications, achieving superior performance while maintaining biological interpretability. To bridge the gap between in silico models and clinical utility, future research must prioritize the standardization of multi-center data and the development of robust frameworks capable of integrating emerging modalities, such as the gut microbiome-metabolome axis, which is pivotal in GI oncology. Future research directions should include the development of dynamic integration frameworks and multimodal foundation models that automatically adapt fusion strategies based on data characteristics (35–37). Addressing hurdles in prospective clinical validation and regulatory compliance will be crucial for transforming these DL-based multi-omics tools into reliable clinical decision-support systems, ultimately improving biomarker discovery and enhancing precision medicine approaches in GI cancers (15).

## Applications of deep learning techniques in GI cancer research

DL techniques have emerged as a transformative approach for identifying clinically significant biomarkers in complex multi-omics datasets for studying GI cancers. Their applications span across diverse molecular layers, including genomics, transcriptomics, proteomics, metabolomics, and radiomics, each providing unique perspectives on tumor biology. The following sections detail these applications; each subsection features a summary table of representative studies and their methodologies to provide a comprehensive overview of the field.

### Genomics-based biomarker discovery

Genomic alterations, such as somatic mutations, copy number variations (CNVs), and DNA methylation changes, serve as critical drivers in the development and progression of GI cancers (38). The vast complexity and scale of genomic data present substantial analytical challenges that conventional methods often struggle to address. DL approaches have shown remarkable potential in mining these intricate genomic datasets to identify biomarkers with clinical utility for early detection, risk assessment, and personalized treatment strategies (39).

CNNs have been particularly effective in analyzing genomic data, where raw sequence information or variant calls are transformed into structured matrix representations. These models, by encoding genomic variant data as structured matrices or sequences, excel at detecting spatial patterns in mutation profiles that correlate with specific molecular subtypes or clinical outcomes. For example, CNN-based analyses of TCGA datasets have successfully classified GI cancers into distinct molecular subgroups with significant prognostic signatures (40).

Autoencoder architectures have become indispensable tools for genomic data analysis, addressing the inherent challenges of high dimensionality and noise in sequencing datasets. Variational and denoising autoencoders have demonstrated particular success in extracting biologically meaningful latent features from whole-genome and exome sequencing data. These compressed representations not only facilitate downstream analyses but also have strong correlations with critical clinical endpoints such as overall survival and treatment response (41).

RNNs have opened new avenues for studying temporal genomics processes. The ability of these methods to model sequential dependencies has been valuable in analyzing DNA methylation dynamics across tumor progression, identifying epigenetic silencing patterns of tumor suppressor genes, and characterizing methylation-based molecular subtypes (42).

The interpretability of DL models has undergone significant advancements through techniques such as SHapley Additive exPlanation (SHAP) analysis and integrated gradients, which provide crucial insights into the genomic features driving model predictions, as well as potential biomarkers (43, 44). These methods have helped bridge the gap between complex model architectures and clinically actionable findings, revealing mutation clusters and regulatory elements with potential biomarker utility. As genomic databases continue to expand and sequencing technologies become more accessible, DL techniques are expected to play an increasingly central role in translating genomic data into clinically relevant biomarkers for GI cancers. **Table 1** provides a curated summary of seminal studies applying these techniques across various GI cancer types, detailing model architectures, validation approaches, and clinical implications.

**Table 1:** Deep learning applications in genomic, transcriptomic, epigenomic, or related multi-omics biomarker discovery for GI cancers.

Application	Method	Data	Main findings	Ref.
Pan-cancer, including GI cancers Biomarker prediction of MSI and homologous recombination deficiency (HRD)	Multiple instance learning DL	Genomics (TCGA, CPTAC)	While developed as a pan-cancer model, this framework demonstrates particular utility in GI cancers by simultaneously predicting MSI and HRD status from somatic mutation profiles. This multimodal integration enables more precise biomarker identification than mutation analysis alone, offering a comprehensive approach to assess therapeutic vulnerabilities in GI cancers.	(45)
GI cancers Subtype discovery	Transformer-based subtype-former model	Multi-omics (TCGA)	This study developed a transformer-based multi-omics integration framework to accurately classify GI cancer subtypes. It enables the discovery of robust, biologically interpretable biomarkers with enhanced specificity for precision oncology applications.	(46)
GI cancers Epigenomic biomarker discovery	CNN and DNN architectures	DNA methylation (TCGA)	This work employs DL to analyze DNA methylation profiles, identifying epigenetic biomarkers that accurately discriminate cancerous tissue from normal tissue, classify molecular subtypes, and correlate them with oncogenic pathway activity. The model demonstrates the clinical utility of methylation-based epigenetic signatures for both diagnostic and prognostic applications in GI cancers.	(47)
GI cancers Biomarker discovery Drug response prediction	Transformer (DeePathNet)	Genomics (ProCan-DepMapSanger, CCLE, TCGA)	This work developed a transformer-based DL network that integrates genomic alterations with pathway activity profiles to significantly enhance biomarker discovery accuracy in GI cancers.	(48)
Digestive system tumor (DST) Classification Early- and late-stage diagnosis	Graph convolutional networks (GCNs)	Multi-omics (TCGA)	This research utilizes multi-omics GCNs for DST classification and early- to late-stage diagnosis, addressing the significant global challenge posed by these prevalent neoplasms.	(49)
DST Subtyping	GCNs Self-encoding model K-means algorithm	Multi-omics (TCGA)	MSDST leverages GCNs and k-means clustering to identify molecularly distinct tumor subtypes through integrative analysis of multi-omics data. This approach provides a computational framework for precision oncology decision-making in GI cancers.	(11)
DST Prognosis prediction Drug response prediction	Graph transformer (GA) Graph attention network (GAN) View correlation discovery network (VCDN)	Multi-omics (TCGA, GDSC)	The multi-omics fusion graph attention network leverages attention-based multi-omics fusion to predict survival outcomes and drug response in DST, demonstrating enhanced predictive accuracy and clinical utility for personalized oncology.	(50)
Gastric cancer (GC) Classification	Deep feature selection	Multi-omics (TCGA)	This study classifies GC using deep feature selection, driven by its high incidence and the advances in high-throughput genomic technology.	(51)
GC Survival prediction	DL-based integration modules	Multi-omics (TCGA)	This work demonstrates the power of DL to integrate multidimensional genomic profiles with clinical covariates for survival prediction in GC. It identifies novel prognostic genomic biomarkers and establishes a DL framework with superior stratification accuracy, providing compelling evidence for implementing DL-driven precision oncology approaches in GI cancers.	(52)
GC Survival stratification	Bidirectional deep neural networks (BiDNNs)	Multi-omics	This study develops a novel survival prediction framework utilizing BiDNNs to integrate multi-omics data (transcriptomics, epigenomics) for precise survival stratification in GC patients.	(53)
Colon cancer (CC) Prognosis prediction	DL integration framework	Multi-omics (TCGA)	This research shows that DL-based integration of multi-omics profiles and clinical variables significantly enhance prognostic accuracy in CC.	(38)
CC Survival stratification	DL-based multi-omics fusion	Multi-omics (TCGA)	This study develops an integrative DL framework that combines transcriptomic (RNA-seq) and epigenomic (DNA methylation) profiles to stratify CC patients into distinct survival groups. These findings enhance personalized prognostic accuracy and reveal key biological mechanisms underlying cancer progression.	(12)
Colon adenocarcinoma (COAD) Survival prediction	DNNs	Multi-omics (TCGA)	This study develops a DL framework that integrates multi-omics data (genomics, transcriptomics) with clinical variables to predict survival outcomes in COAD. The model identifies robust prognostic biomarkers across molecular layers and enables risk stratification with potential clinical utility for personalized therapeutic decision-making.	(54)
CRC Subtype classification	DL-based subtype classifier	Multi-omics	The work developed a DL framework that leverages multi-omics data to classify molecular subtypes of CRC and provide clinically relevant cancer cell line matches, advancing multi-omics-guided precision oncology for GI cancers.	(55)
CRC Prognostic risk prediction	DL combined with LASSO and SVM	Multi-omics (TCGA)	This work developed a DL-based multi-omics risk model for CRC, with a focus on epithelial–mesenchymal transition (EMT)-related genes, identifying novel prognostic biomarkers and achieving superior risk stratification.	(56)
Hepatocellular carcinoma (HCC) Survival prediction	DL ensemble	Multi-omics (TCGA)	The learning framework identifies prognostic biomarkers spanning multi-omics layers (RNA-Seq, miRNA-Seq, and methylation), achieving superior prediction accuracy while providing mechanistic insights into cancer progression and enabling precision risk stratification for therapeutic decision-making.	(57)
HCC Prognosis prediction	BiDNNs	Multi-omics (TCGA)	This study developed a BDNN framework that integrates DNA methylation and mRNA expression data to predict clinical outcomes in HCC. This approach not only achieves superior prognostic accuracy but also identifies novel, biologically interpretable genomic biomarkers of cancer progression.	(58)

### Transcriptomics and epigenomics in GI cancer biomarker discovery

Transcriptomic profiling, which involves both mRNA and non-coding RNA expression patterns, provides critical insights into the dynamic molecular activity of GI cancers and their responses to microenvironmental cues. The dysregulation of gene expression programs and epigenetic modifications, including DNA methylation and histone alterations, has been firmly established as a hallmark of GI cancer progression (59).

RNNs and their LSTM variants have demonstrated efficacy in analyzing sequential transcriptomic data, modeling temporal expression dynamics to predict cancer staging and metastatic potential (60). More recently, Transformer-based models have enhanced this performance through their ability to process entire transcriptomes simultaneously via self-attention mechanisms, significantly improving performance using large-scale RNA sequencing (RNA-seq) datasets (61).

In the epigenomic domain, specialized DL architectures have been developed to analyze chromatin accessibility profiles (e.g., ATAC-seq), histone modification patterns, and genome-wide methylation states. Models such as DeepCpG and epigenetic CNNs have shown remarkable accuracy in predicting methylation landscapes and identifying regulatory elements crucial for oncogenic gene expression programs in cancer cells. Recent advances in multi-modal integration frameworks, employing either branched network architectures or cross-modal attention layers, have enabled the systematic investigation of coordinated transcriptomic and epigenomic alterations driving tumorigenesis (10).

The incorporation of attention-based models, including self-attention and cross-modal attention layers, has been particularly transformative, allowing models to dynamically weigh the contribution of individual transcripts or epigenetic marks to prediction outcomes (62). These interpretable architectures not only generate biologically plausible biomarker candidates but also achieve superior predictive performance by highlighting molecular features with established roles in cancer biology. **Table 1** summarizes seminal studies applying these DL approaches to transcriptomic, epigenomic, or related multi-omics biomarker discovery in GI cancers.

### Proteomics and metabolomics in GI cancer biomarker discovery

Proteomic and metabolomic profiling provides critical functional insights into the downstream molecular consequences of genomic and transcriptomic alterations. Unlike static genomic markers, metabolites reflect the real-time phenotype of the tumor and its interaction with the gut microbiome (63, 64). Dysregulated protein expression, post-translational modifications, and metabolite reprogramming are hallmarks of cancer progression and therapeutic resistance, making these omics layers particularly valuable for biomarker discovery (65, 66). DL technology has emerged as a powerful approach to decoding complex patterns within these high-dimensional datasets for biomarker identification (67).

In proteomics, CNNs and autoencoder architectures have exhibited strong performance in analyzing mass spectrometry and antibody protein microarray data. These models excel at identifying protein signatures associated with clinical outcomes, such as distinguishing early-stage from late-stage CRC on the basis of differential protein expression patterns (9). Autoencoders, in particular, enable efficient dimensionality reduction while preserving biologically relevant features for downstream analysis.

GNNs have been especially valuable in proteomic studies because of their ability to model protein–protein interaction networks. By analyzing topological features within these biological networks, GNNs can prioritize hub proteins that play central roles in oncogenic signaling pathways. These network-based biomarkers often exhibit greater biological relevance and clinical utility than individual protein markers do because of their functional relevance (8).

Metabolomic profiling, which captures the dynamic biochemical state of cancers, has benefited significantly from VAEs and deep belief networks (DBNs). These architectures effectively model the nonlinear relationships between metabolite concentrations and disease phenotypes, enabling the identification of metabolic signatures linked to cancer cachexia, chemoresistance, and disease recurrence.

The integration of proteomic and metabolomic data through multi-input neural networks has further enhanced biomarker discovery by revealing functional interactions across molecular layers. These approaches capture the complex interplay between proteins and metabolites, providing a more comprehensive understanding of cancer biology. **Table 2** highlights key studies that have employed DL techniques to identify proteomic and metabolomic biomarkers in GI cancers.

**Table 2:** Representative studies using deep learning for proteomic and metabolomic biomarker discovery in GI cancers.

Application	Method	Data	Main findings	Ref.
Disease feature extraction for classification	Deep Autoencoders CNNs	Synthetic and real metabolomics datasets (method-focused, experimental)	While maintaining a broad range of applications, this study systematically evaluates DL applications in metabolomics pipelines, with specific validation in cancer biomarker discovery. The experimental framework demonstrates DL’s unique capability to extract latent metabolite patterns that significantly enhance biomarker identification accuracy.	(68)
Cancers Subtype classification Drug response prediction	Transformer-based DL (DeePathNet)	Multi-omics, including proteomics (ProCan-DepMapSanger, CCLE, TCGA)	DeePathNet is an interpretable DL framework that integrates prior biological pathway knowledge to analyze multi-omics data, demonstrating superior performance in both cancer type classification and drug response prediction.	(69)
GI cancers Biomarker discovery	DL-based biomarker analysis	Multi-omics, including proteomic and metabolomic datasets	This study employs DL to systematically discover proteomic and metabolomic biomarkers across multiple cancer types, with particular emphasis on GI cancers. By integrating MS-based omics data with advanced neural works, this work identifies disease-specific biomarker panels and reveal the critical role of metabolite–protein interactions in enhancing the precision of GI cancer diagnosis and monitoring.	(70)
GI cancers Biomarker discovery	Autoencoder Deep transfer learning	Multi-omics, including liquid chromatography–mass spectrometry, gas chromatography–mass spectrometry, and nuclear magnetic resonance (NMR) datasets	While initially validated in HER2+ samples, the method demonstrates generalizability to GI cancers through DL-based metabolomic integration. The framework identifies key metabolic features (e.g., phosphatidylcholine, β-alanine) as diagnostic biomarkers, establishing a robust approach for early-stage, multi-platform biomarker discovery in precision oncology.	(71)
GI cancers Cancer diagnosis	Random forest (RF) Support vector machine (SVM) Logistic regression (LR) k-nearest neighbors (k-NN) Naïve Bayes (NB)	Selected reaction monitoring–mass spectrometry data (1,008 samples)	This study develops a DL model for cancer diagnosis by analyzing quantitative proteomic profiles. The model achieves high diagnostic accuracy (AUC = 0.9472) and identifies clinically relevant protein biomarkers, highlighting its potential as a proteome-based screening tool for GI cancer diagnostics.	(72)
GI cancers Subtyping	Federated DL	Multiple decentralized proteomic datasets	This study implements federated learning to develop DL models across distributed proteomic datasets while preserving patient data privacy. It successfully identifies clinically relevant cancer subtypes from proteomic profiles, demonstrating that privacy-preserving AI can extract meaningful biomarkers for GI cancer diagnostics and classification.	(73)
GI cancers Subtyping Prognosis prediction	Autoencoder-based DL	Multi-omics, including proteomics and metabolomics	This study develops an integrative framework that combines proteomic, metabolomic, and other omics data to generate latent feature representations for cancer stratification. When applied to GI cancers, it identifies clinically relevant multi-omics biomarker patterns that significantly correlate with patient outcomes, demonstrating DL’s capability to derive biologically meaningful signatures from complex, multi-layered molecular data.	(74)
GI cancers Recurrence prediction Biomarker discovery	Attention-based DL plus pathway alignment	Multi-omics, including proteomics	DeepKEGG integrates KEGG pathways knowledge as biological priors in DL to identify interpretable proteomic biomarkers for cancer recurrence. The model improves both prediction accuracy and biological explainability, revealing pathway-level proteomic features with significant prognostic value in GI cancers.	(75)

### Radiomics and pathomics: multi-scale imaging biomarkers in GI oncology

Radiomics has revolutionized GI cancer research through its ability to extract high-throughput quantitative features from medical images, enabling non-invasive characterization of cancer phenotypes at the macro-scale. This approach has demonstrated significant clinical value in cancer grading, molecular classification, and treatment response evaluation across various GI cancers (76–78). DL techniques, particularly CNNs, have expanded radiomics beyond traditional feature engineering through autonomously learning complex imaging signatures that correlate with clinically relevant end points (18, 19). Parallel to these macro-scale advancements, the integration of pathomics—the deep learning-based analysis of micro-scale histopathological whole-slide images (WSIs)—has provided a more granular perspective on the tumor microenvironment.

Specifically, CNN-based architectures have shown remarkable success in analyzing standard-of-care imaging modalities, including computed tomography (CT), magnetic resonance imaging (MRI), and positron emission tomography (PET) scans. These models effectively capture subtle variations in textural patterns, morphological features, and contrast dynamics that correspond to underlying histopathological characteristics or specific genomic alterations. Notably, DL models have achieved clinically relevant performance in predicting MSI status and KRAS mutations directly from routine radiological examinations, offering a potential alternative to invasive molecular testing (6).

The emerging field of radiogenomics has further enhanced biomarker discovery by establishing direct correlations between imaging phenotypes and molecular profiles. Compared with that of single-modality approaches, advanced hybrid architectures that integrate CNN-processed imaging data with genomic or epigenomic information have demonstrated superior predictive accuracy, particularly in prognostic stratification and therapeutic decision-making (79). These multi-scale approaches bridge the gap between gross tumor morphology and molecular dysregulation.

Recent innovations have incorporated transformer architectures and attention mechanisms into radiomic pipelines, addressing several key limitations of conventional CNN approaches. These techniques improve model interpretability by identifying clinically relevant regions of interest while capturing long-range spatial dependencies within imaging sequences. The resulting models not only achieve high predictive performance but also provide biological insights by linking specific imaging features, the most informative regions of interest (ROIs), to molecular pathways (80).

Despite these advances, ongoing challenges remain, including the need for standardized imaging protocols across institutions and the scarcity of comprehensively annotated datasets. Nevertheless, the scalability of DL approaches and their ability to extract meaningful information from routine clinical imaging position multi-scale imaging integration as a transformative modality in GI cancer biomarker research. **Table 3** summarizes representative studies applying these advanced techniques to GI oncology.

**Table 3:** Deep learning applications in macro-scale radiomics and multimodal imaging for GI cancer biomarker discovery.

Application	Method	Data	Main findings	Ref.
Pan-cancer, including GI cancers Biomarker prediction	Multiple CNN models	Multi-omics, including whole-slide images (WSIs) (TCGA)	This large-scale study demonstrates that DL models can accurately predict genetic alterations, transcriptomic profiles, proteomic signatures, and metabolic biomarkers directly from H&E-stained histology images across 32 cancer types. The findings establish that routine pathology slides contain rich molecular information that can be decoded through computational approaches.	(81)
GC Tumor mutational burden (TMB) prediction	Multimodal DL	Multimodal data, including histopathological images (TCGA)	This study develops a multimodal DL framework to predict TMB from histopathological images in GC. The integrated modal, which combines histopathological images with molecular omics data, demonstrates superior performance compared to image-only approaches, establishing an effective paradigm for computational TMB assessment.	(59)
GC Lymph node metastasis prediction	DL radiomic nomogram	CT images	This study developed a DL-powered radiomic nomogram that quantifies lymph node metastasis risk in locally advanced GC. By integrating multiparametric CT radiomics with clinical biomarkers, the model significantly improves preoperative modal staging accuracy, enabling more precise surgical decision-making.	(14)
GC Peritoneal recurrence and survival prediction	Multitask DL	Preoperative CT images	This study develops a multitask DL model that simultaneously predicts peritoneal recurrence and overall survival from preoperative CT images in GC patients. The model predicted peritoneal recurrence and disease-free survival independently of clinicopathological variables accurately for identifying patients who may benefit from intensive treatment.	(82)
CC MSI status prediction Heterogeneity quantification	Deep CNN with feature attention	H&E-stained histology images (TCGA)	This work developed a DL framework that analyzes routine histopathological images to simultaneously predict MSI status and quantify intra-tumoral heterogeneity in CC, providing spatial heterogeneity maps that correlate with genomic instability patterns.	(83)
CRC Mutation and pathway activity prediction	Weakly supervised deep CNN	Histology images (TCGA)	This work built a DL model to predict key driver mutations and pathway activation status from WSIs. Validated across two independent cohorts, the model demonstrated that computational pathology can reliably infer genomic alterations from tissue morphology.	(84)
CRC MSI status prediction	Multimodal DL	Multi-omics, including H&E staining images (TCGA)	This study developed a multimodal DL framework that integrates histopathological images with molecular profiling data to predict MSI status in CRC. The combined approach achieved superior performance compared to image-only models, demonstrating the value of multimodal integration for immunotherapy-relevant biomarker detection and decision-making.	(85)
CRC MSI prediction	MSINet	H&E-stained WSIs (TCGA)	This study developed a CNN that accurately predicts MSI status from routine H&E-stained CRC histology slides. The model demonstrated robust performance across multiple validation cohorts, revealing that DL can extract reliable imaging biomarkers indicative of genomic instability patterns.	(86)
CRC TMB prediction	Multi-modal DL	Multi-omics, including histopathological images	This study constructs a multimodal DL model that integrates histopathology images with clinical variables to predict TMB in CRC. The model achieved significant predictive performance, demonstrating the feasibility of non-invasive TMB estimation from routine diagnostic materials.	(21)
CRC Prognosis prediction Molecular profile inference	Multi-omics multi-cohort assessment ML platform	Multi-omics, including histopathology images (TCGA)	This DL model establishes biologically interpretable associations between histopathological patterns and molecular profiles, accurately predicting both gene expression levels and DNA methylation status directly from routine H&E slides. This approach enables comprehensive multi-omics inference from standard pathology specimens.	(39)
CRC Prognostic biomarker discovery	U‐Net classification network TransMIL model	Multi-omics, including pathological images (TCGA)	This work developed a comprehensive DL framework that systematically identifies histopathological image features predictive of both genomic alterations and clinical outcomes in CRC. This approach reveals novel and interpretable image–genomics correlations that may serve as robust prognostic biomarkers.	(87)
CRC Prognosis prediction and stratification	End-to-end deep neural network	Histopathological images from multiple centers	This study demonstrates that DL models trained on digital pathology images achieve high accuracy in predicting CRC outcomes. The model shows comparable performance to board-certified pathologists while enabling non-invasive discovery of novel prognostic biomarkers.	(88)
CRC Prognosis prediction	CNNs	H&E-stained sections (TCGA)	This work developed a DL model that analyzes histology images to predict CRC patient outcomes. Validation across independent datasets revealed consistent performance and identified spatial histological features significantly associated with overall survival.	(89)
CRC Prognosis and therapy response prediction	Multistain DL model	Multistain histopathology images (multi-center)	This study designed a multimodal DL framework that integrates H&E- and IHC-stained histopathology images to predict therapy response and survival outcomes in CRC. The multistain approach outperformed single-modality models, demonstrating enhanced biomarker discovery through multimodal image fusion.	(90)
CRC Tumor–stroma ratio (TSR) and therapy outcome prediction	Multitask DL model	Preoperative CT images	This multitask DL model simultaneously predicts the TSR and treatment response from preoperative CT scans with high accuracy. The framework identified radiomic features that significantly correlated with stroma-related prognostic markers, enabling comprehensive pretreatment risk assessment.	(91)
Rectal cancer Distant metastasis prediction	Deep residual learning Cox proportional hazard model	MRI scans from multi-center rectal cancer cohorts	This work demonstrates that radiomic features extracted from pre-treatment MRI scans can predict distant metastasis risk in rectal cancer with high accuracy. The DL model achieved superior risk stratification compared to clinical staging, supporting its use for personalized surveillance planning.	(92)
Rectal cancer Prognosis prediction	CNN-based DL	MRI scans	This study developed a DL model that analyzes preoperative MRI scans to predict treatment outcomes in rectal cancer with high accuracy. The model identified radiomic features that were significantly associated with disease-free survival, providing quantitative biomarkers for clinical decision-making.	(93)
Liver cancer Prognosis prediction	CNN-based DL (PathFinder)	Histology images	PathFinder is an interpretable DL framework that identifies prognostic tissue biomarkers by analyzing spatial histopathological patterns (e.g., necrosis architecture, tumor–stroma distribution). The model achieves significant prediction accuracy for cancer outcomes while providing feature importance maps for clinical interpretation.	(94)

### Multi-omics and cross-modality integration in GI cancer biomarker discovery

The integration of multi-omics data represents a paradigm shift in biomarker discovery, enabling a systems-level understanding of GI cancers by combining genomic, transcriptomic, proteomic, metabolomic, and multi-scale imaging information. DL methods have emerged as a powerful approach for this integration and are capable of learning complex cross-modal relationships that traditional methods often miss (57, 95). These models excel at identifying both vertical relationships within omics layers and horizontal connections across different data types, providing a more comprehensive view of cancer biology.

Early integration approaches focused on concatenating features from different omics layers into a single input matrix for processing through fully connected DNNs or CNNs. While straightforward, these methods often encounter challenges with high dimensionality and require careful feature selection. Intermediate integration strategies using modular architectures such as multi-branch autoencoders or transformer-based models have achieved superior performance by first learning modality-specific features before combining them. Recently, the adaptation of multimodal foundation models for intermediate fusion has shown promise in capturing deeper latent correlations across disparate data scales. These approaches have demonstrated particular success in predicting clinical outcomes such as patient survival in CRC and recurrence risk in patients with gastric cancer (4).

Late integration methods train separate models on individual omics modalities and combine their predictions through ensemble techniques, offering greater flexibility and robustness to missing data, which is suitable for real-world clinical datasets. GNNs and cross-modal attention mechanisms have been especially valuable for capturing relationships between molecular and imaging data, especially in radiogenomic and pathogenomic applications (59, 85). These approaches not only increase predictive accuracy but also support biological interpretability by revealing meaningful connections across data types.

## Critical challenges and future directions

### Data limitations and generalizability

A primary obstacle is the scarcity of large-scale, comprehensively annotated multi-omics datasets. A critical analysis of the current literature reveals an over-reliance on TCGA. While invaluable, TCGA data may not fully represent real-world patient diversity, and models trained solely on this dataset are prone to overfitting. In the context of GI cancers, a specific challenge is the significant intratumoral heterogeneity and the temporal evolution of the tumor microenvironment, which static public datasets fail to capture. Furthermore, TCGA lacks comprehensive radiological imaging for many cohorts.

Future direction: To ensure clinical utility, future studies must prioritize the establishment of multi-center longitudinal biobanks that integrate spatial transcriptomics and serial imaging. This will allow DL models to transition from static predictions to dynamic monitoring of tumor progression and therapy resistance. External validation on diverse ethnic populations is essential to demonstrate model robustness.

### Interpretability and clinical trust

The “black box” nature of many sophisticated architectures creates barriers to understanding the biological rationale behind predictions, limiting clinician trust. While emerging approaches incorporating attention mechanisms and explainable AI (XAI) techniques (e.g., SHAP, integrated gradients) show promise, significant work remains to establish standardized frameworks for biological interpretation [49]. For GI oncology, interpretability must extend beyond abstract heatmaps to identify actionable biological entities, such as specific microbial species in the gut microbiome-metabolome axis or metabolic vulnerabilities in gastric cancer cells.

Future direction: Future efforts should focus on “biologically-informed deep learning”, where prior knowledge from biological pathways (e.g., KEGG, Reactome) is embedded into the neural network architecture. This ensures that the hidden layers represent tangible biological processes, making the “black box” transparent to clinicians.

### Computational and privacy constraints

The training of complex models on high-dimensional multi-omics datasets demands substantial resources. Furthermore, data privacy regulations restrict access to the large, diverse datasets needed for robust model development (96, 97). Federated learning frameworks offer a promising solution to overcome data sharing barriers by enabling privacy-preserving collaborative model development across institutions (98, 99). Additionally, the emergence of multimodal foundation models (100–102) provides a transformative approach to knowledge transfer, potentially reducing the reliance on massive labeled datasets by leveraging self-supervised pre-training on diverse biomedical corpora.

Future direction: The field should move towards “small-data learning” through transfer learning from biomedical foundation models, allowing high-performance biomarker discovery even in rare GI cancer subtypes where large cohorts are unavailable.

## Conclusions

Gastrointestinal cancers present significant global health challenges, underpinned by complex molecular mechanisms that demand robust, clinically actionable biomarkers. Intelligent computing methodologies, particularly deep learning, coupled with multi-omics and imaging data integration, offer transformative potential for biomarker discovery—enabling improved risk prediction, early diagnosis, patient stratification, and therapy response assessment. However, the field must transition from proof-of-concept studies on public datasets to rigorous validation in clinical settings. Crucially, the next phase of development must address hurdles in prospective clinical trial design and regulatory approval processes (e.g., FDA/NMPA), ensuring that these tools meet the stringent standards for clinical decision support systems (CDSS). Future efforts should focus on enhancing model interpretability, addressing data heterogeneity through standardized multi-center protocols, and validating biomarkers in prospective multi-center trials. By evolving toward biologically-interpretable architectures and validating them through standardized multi-center protocols, deep learning will bridge the gap between molecular insights and improved patient outcomes, ultimately contributing to more effective, individualized gastrointestinal cancer management.
